# Remembering the null hypothesis when searching for brain sex differences

**DOI:** 10.1186/s13293-024-00585-4

**Published:** 2024-02-09

**Authors:** Lise Eliot

**Affiliations:** Stanson Toshok Center for Brain Function and Repair, Chicago Medical School, Rosalind Franklin University of Medicine & Science, North Chicago, IL USA

## Abstract

Human brain sex differences have fascinated scholars for centuries and become a key focus of neuroscientists since the dawn of MRI. We recently published a major review in *Neuroscience and Biobehavioral Reviews* showing that most male–female brain differences in humans are small and few have been reliably replicated. Although widely cited, this work was the target of a critical Commentary by DeCasien et al. (Biol Sex Differ 13:43, 2022). In this response, I update our findings and confirm the small effect sizes and pronounced scatter across recent large neuroimaging studies of human sex/gender difference. Based on the sum of data, neuroscientists would be well-advised to take the null hypothesis seriously: that men and women’s brains are fundamentally similar, or “monomorphic”. This perspective has important implications for how we study the genesis of behavioral and neuropsychiatric gender disparities.

## Plain language summary

The idea that men and women have fundamentally different brains has prevailed among scientists and non-scientists alike. MRI studies have been grappling with this for more than three decades but yet to find distinct features that define the “male brain” versus “female brain” across the human species. In seeking the causes of gender disparities in behavior and mental health, scientists need to look well beyond brain structure.

## Highlights

- Current institutional policies have elevated the study of binary sex/gender difference over other dimensions of individual difference.
- Once brain size is accounted for in MRI analyses, sex/gender differences are small and not reliably found across recent large studies of regional human brain volumes.
- Given this variability and the multifactorial origins of human brain sex/gender differences, researchers should exert greater caution when interpreting their relevance to gender behavioral and health disparities.

Men and women are different: physically, psychologically, socially, economically, and politically. Across academic fields, researchers find gender gaps in every domain of human existence. The question for biologists, and neuroscientists in particular, is: why? Do these differences emanate from two categorically distinct (“dimorphic”) types of brains? Or are they reflections of similar brains reacting to different physical, psychological, social, economic, and political circumstances?

Most human neuroimaging to-date has been focused on the former question: identifying a set of fixed, categorical differences that will reliably distinguish men’s and women’s brains across the species. In 2021, my colleagues and I published a large synthesis in *Neuroscience and Biobehavioral Reviews (NBBR)* covering three decades of brain imaging studies to evaluate the evidence for such reliable, categorical sex/gender^1^ differences [1]. We spent several years repeatedly searching this massive literature across MRI studies of brain structure, function, and connectivity, and also included post-mortem structural and histological studies. Our analysis was based on 616 mostly primary research articles, including 33 meta-analyses. We tabulated all of the large and highly cited studies that our search uncovered, but focused on the largest studies and meta-analyses in formulating our conclusions.

Here’s what we found: men and women’s brains clearly differ on one measure—men’s brains are some 11% larger in total volume, a value replicated across several large UK studies. While a statistically large effect (*d* = 1.31 in the largest study to-date; [2]), brain volume nonetheless overlaps by 51% between female and male distributions, so not a “categorical” difference or “dimorphism”—that is, like the difference between ovaries and testes, or the tail of a peacock versus peahen.

In their critique, DeCasien et al. agreed about the inappropriateness of the term “dimorphism”, however, they challenged a major portion of our analysis, suggesting that the single large study by Williams et al. [2], which included over 40,000 participants, essentially supplants all the prior findings and represents the ground truth or “biological reality” of human male–female brain differences (to quote the title of their paper). Specifically, they challenged our conclusion about the poor reproducibility of sex/gender differences in regional brain volumes—that is, specific cortical areas and subcortical structures claimed to be larger in men or women, even after total brain volume is accounted for.

The latter control is important, because men average some 18% heavier and 9% taller than women [3], so larger brain volume would be predicted based on the difference in limb size, muscle mass, and other innervated tissues. In fact, we observed that the 11% average difference between women and men’s brain volume is considerably smaller than the sex/gender size difference in other internal organs such as the heart (17%), lungs (23%), and kidneys (19%), [4]. Interestingly, it was a lot harder to find sex/gender difference data for these other organs in the scientific literature, compared to many hundreds of studies comparing men and women’s brain size, a topic of ancient fascination that speaks to the issue of sexism in this area of research.

Not surprisingly, larger brains are associated with larger volumes of every CNS compartment: ventricles, white matter, cortical gyri, basal ganglia, diencephalon, cerebellum, and brainstem. So the real question is whether specific structures are proportionally larger in males or females, which animal research tell us is pertinent to behavioral sex differences. This was first dramatically demonstrated in songbirds, in which several forebrain nuclei are at least 800% percent larger in males and associated with male-predominant courtship singing [5]. But based on the 33 studies of subcortical and 25 studies cortical structures we reviewed, no structures come close to this difference in humans, once individual brain or head size is controlled for: the largest differences amount to about 2% larger amygdala and putamen in men, and 1.3% larger nucleus accumbens and 2.1% larger middle frontal gyrus in women. And even these modest findings are not fully reliable, with the magnitude and even direction of many sex/gender differences turning out to be highly sensitive to the complex imaging analysis pipeline, including various bias corrections, spatial normalization, atlas registration and structural segmentation, and controls for individual brain size.

DeCasien et al. [6] argue that we can ignore nearly all of these many dozen studies, based on their small size and varying controls for brain size. We do not necessarily disagree about this, since our conclusions about regional cortical volumes were based on the dozen largest, most recent studies (Table 3 in [1]). Nonetheless, it was important to tabulate all the extant findings, since many very small studies with strong, unreplicated claims about sex/gender difference continue to be widely cited (e.g., [7]).

The importance of large samples for analyzing sex/gender differences [8] and especially, behavioral correlates of brain MRI measures [9] has now been well-established. Thus, Table 1 of the present paper lists the six largest studies [10–14, 20] of male/female regional cortical volume differences, including Williams et al. [2, 15] that was published after our *NBBR* review. Although there is clearly some replication in the direction of sex/gender difference in certain cortical areas (e.g., medial occipital, inferior temporal, superior parietal), the overall picture is diverse, and not what would be found in studies of, say, height, facial hair, or genital morphology. Rather, across these several large brain studies, sex/gender differences are inconsistent in many cortical areas, their effect sizes are generally small (Cohen’s *d* < 0.20), and they range substantially across studies. Although small effects are not necessarily unimportant, they do contribute to variability [16], since smaller differences are more likely to fluctuate across the noise of different methods and populations. Indeed, we dedicated considerable discussion in the *NBBR* paper to key methodological factors that could contribute to this variance, especially volume- versus surface-based segmentation methods and proportional versus covariance methods of controlling for brain size. Thus, it is difficult to accept that neuroimaging researchers have finally, as of 2021, settled on the optimal methods (or “best practices”, as DeCasien et al. phrase it) for processing brain images, controlling for individual size, and automatically segmenting them into component structures [17]—thereby uncovered the ground truth of brain sex/gender difference. On the contrary, such methods are likely to continue improving, to more closely approximate the “gold standard” of manual segmentation by human neuroanatomists (e.g., [18]), and to add other sex/gender-related covariates, such as head shape [19], whose influence on individual differences in regional brain volumes have thus far been overlooked.

For now, I note that that the volumetric sex/gender differences asserted by DeCasien et al. [6] have not held up well in large recent datasets, as their own Fig. 1D shows. Their figure depicts whole brains with colorized areas for sex/gender differences found in “one, two, or all three” cohorts they analyzed (from the US, the UK and Germany), ranging in size from about 1000 to 2800 participants (German data from [12], US and UK data from [13]). However, only a small minority of regions differed significantly in the same way across all three cohorts. Moreover, these regions do not match the much larger study (*N* > 40,000) they praise by Williams et al. [2]. Indeed, the direction of sex/gender effects they depict in these three cohorts is opposite that reported by Williams et al. [2] for many major cortical gyri including: superior frontal, inferior frontal, orbitofrontal, pre-central, right inferior parietal and precuneus, superior temporal, parahippocampal, anterior cingulate, and insula (see Table 1). With regard to cerebellar gray matter, DeCasien et al.’s Fig. 1D also differs diametrically from Williams et al. [2], who reported greater volumes in women for 82 percent of cerebellar regions, whereas DeCasien et al. show most cerebellar regions larger in *men*. Moreover, even Williams et al. failed to replicate 38% of the volumetric sex/gender findings from an earlier study of over 5000 participants [20] using the same UK Biobank database (see [15], Suppl. Figure S3). Although many will consider a 62% replication rate good and argue that the discrepancies could be due to slight methodological differences between the two studies, the 38% non-replicated findings could also reflect a true change in sex/gender findings for this sample population when it was expanded eightfold.

**Table 1:**
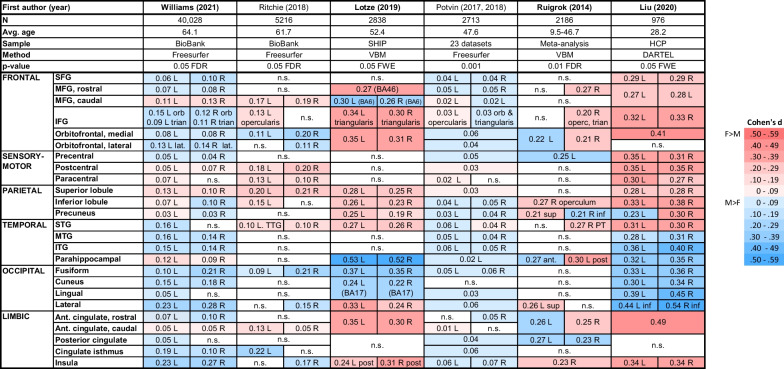
Sex/gender differences in human regional cerebral cortical volumes reported across recent large studies

Tabulation of findings from the six largest studies derived and expanded from Eliot et al. [1] Table 3, with all measures controlled for individuals’ total brain or intracranial volume. Studies are ordered by sample size from largest to smallest. Bolded study names are those highlighted by DeCasien et al. [6]. Each cell lists effect size (as Cohen’s d-values) for the male-female difference in volume of that cortical region, where “L” indicates left hemisphere, “R” indicates right hemisphere, and the absence of either letter indicates a bilateral measure. Although all of these studies were well-powered, the small magnitude of the differences (≤ 2% of structural volume), divergent methods (image pre-processing, global size correction, and cortical segmentation), and true population differences presumably account for the lack of consistency between them

Cell shading: pink = significantly larger in females; blue = significantly larger in males; white (n.s.) = not significant

Abbreviations: ant (anterior); BA (Brodmann’s area); DARTEL (diffeomorphic anatomical registration through exponentiated lie algebra); FDR (false discovery rate); FWE (familywise error rate); HCP (Human Connectome Project, United States); IFG (inferior frontal gyrus); inf (inferior); ITG (inferior temporal gyrus); lat (lateral); MFG (middle frontal gyrus); MTG (middle temporal gyrus); operc (pars opercularis); orb (pars orbitalis); post (posterior); PT (planum temporale); SFG (superior frontal gyrus); SHIP (Study of Health in Pomerania, Germany); STG (superior temporal gyrus); sup (superior); trian (pars triangularis); TTG (transverse temporal gyrus); VBM (voxel-based morphometry)

Despite asserting that large-scale imaging has now identified “highly reproducible” brain sex/gender differences, DeCasien et al. [6] state other contradictory findings as well. Thus, in a section praising the authority of large individual studies, they note that Williams et al. [2] found the hippocampus to be some 1.5% larger in women. But just two paragraphs later, they write that a consensus of other large studies “demonstrates that humans show a highly reproducible spatial pattern of sex differences in regional GMV, including *male-biased* volumes of the putamen, amygdala, *hippocampus,* and temporal pole” [my emphasis]. For the record, Williams et al. [15] published a Supplemental Figure S2 that showed substantial discrepancies between their volumetric sex/gender differences in the hippocampus, pallidum, thalamus and caudate nucleus compared to those reported by Lotze et al. [12] and Liu et al. [13]—the same three cohorts DeCasien et al. [6] synthesize in their Fig. 1D.

Williams et al. [2] were also more cautious in their interpretation, recognizing the “high variability of sex differences” across studies due to numerous methodological and demographic factors. Thus, the “biological reality” that Williams et al. [2] were striving for was not an absolute, species-wide declaration about sex/gender differences in the human brain, but a normative description of the UK population, recognizing the limitations of ethnic distribution and educational attainment in their very large, but not representative Biobank dataset.

Now let us consider the actual size of the sex/gender differences in question. The advantage of large studies is that they have great power to find very small differences, and that is what we are talking about for human brain sex/gender findings. In stark contrast to the 800 percent larger RA (robust nucleus of the arcopallium) and HVC (acronym used as the proper name) nuclei, and the infinitely larger Area X (which does not exist at all in females) in the male zebra finch forebrain [5], the largest of the regional differences between women and men reported by any of the aforementioned human studies is a mere 2%. Similarly, in meta-analyses by own team, we found only 0.6% larger hippocampus (*d* = 0.08, n.s.; [21] and 1.3% smaller amygdala (*d* = 0.20, n.s.; [22] in women, relative to men. Neither effect is out of the noise, which helps explain the considerable jitter in findings across large studies. Given the many processing and normalization steps in the MRI pipeline, it is little wonder that every new segmentation, bias correction, or statistical method can differentially affect brains of different size and shape, creating the cacophony of findings we noted in Eliot et al. [1].

DeCasien et al. focused their critique on our analysis of regional brain volumes, but I will add that Eliot et al. [1] also dove deeply into many other brain features oft-described as “sexually dimorphic”. Most of these, such as the 6% greater gray matter-to-white matter ratio [20, 23] and the higher interhemispheric-to-intrahemispheric connectivity ratio [24] in women are fully attributable to brain size. (Bigger brains have proportionally less gray matter and less efficient interhemispheric connectivity, regardless of sex [25–27].) Moreover, despite these differences in interhemispheric connectivity, meta-analysis of fMRI studies found no significant sex/gender difference in the lateralization of language [28]. Nor, importantly, can the tiny sex/gender difference (*d* = 0.06) in functional asymmetry account for any other cognitive sex/gender differences, according to systematic review and meta-analysis by Hirnstein et al. [29].

In addition to lateralization, we analyzed many dozen fMRI studies that have sought sex/gender differences in localized brain activation, using tasks with well-known gender performance gaps. Across 33 studies using language tasks, 34 using spatial tasks, and seven meta-analyses including dozens of studies using empathy tasks, we found that no task has yielded a reliable sex/gender difference in brain activation (see also [30]). Although many of these studies report some areas of activation that were stronger in either men or women, the lack of reproducibility is likely attributable to false positives, as a meta-analysis of 179 fMRI studies of sex/gender difference has demonstrated [31]. Finally, we showed that 31 recent studies of the structural and functional “connectome” have not found consistent sex/gender differences in global connectivity; although there are hints that the default mode network may be more active in women, such studies have only rarely controlled for brain size, which could influence connectivity differences.

We are not the only ones to observe a profound “literature of contradiction” within the sex/gender difference field [32]. As more and more scientists heed policies to analyze their data by sex, underpowered and statistically mis-analyzed findings are turning out to be common [33]. Although false positives are less of a problem with large datasets such as the UK Biobank, these present their own unique challenges, as such mega samples make it easy to “detect statistically significant interactions, including sex differences, of clinically trivial and meaningless magnitude” [32]. Such findings are perhaps more likely to be reported than other group differences, given mandates by the NIH and other agencies to study sex and the existence of journals, such as this, dedicated to sex difference findings.

Which brings us back to the various sex/gender differences found with structural MRI. It is important to note that most of this research—and all the studies discussed by DeCasien et al.—are based on populations of largely European origin. In particular, the UK Biobank comprised 95% White participants, as compared to a global White population of 12% [34], making the study by Williams et al. [2] unlikely to represent the species as a whole. On the rare occasions when neuroimagers have explicitly attempted to replicate brain sex/gender differences across ethnically dissimilar populations, the reproducibility has been poor (e.g., [35, 36]).

And yet, much of the commentary by DeCasien et al. focuses on evolutionary arguments that presuppose such species-wide brain sex/gender differences. Here they present an uncritical recitation of evolutionary psychological theory, without noting its paternalistic legacy or substantial revision over recent years [37, 38]. Thus, DeCasien et al. anoint a “powerful explanatory framework” to assumptions about male-only competition and a gendered division of labor in deep history, despite more recent evidence challenging dogmas about chaste, uncompetitive females [37, 39] and “man the hunter”[40, 41]. DeCasien et al. also cite select hormone and genetic studies to make the case for innate brain sex/gender organization, despite the weakness of this evidence [42, 43]. Indeed, after decades of research on gonadal hormones, there remains scant consensus on how they influence the human brain at either the prenatal [44], pubertal [45], or menopausal [46, 47] transitions. And with regard to direct genetic influences, the fact that XY individuals with complete androgen insensitivity syndrome appear both psychologically and phenotypically female indicates that genes alone do not “masculinize” the human brain in any behaviorally relevant way [48].

What is missing from this version of “biological reality” is any mention of neuroplasticity: the malleability built into every corner of our nervous system that allows individuals to learn and rapidly adapt to the ambient social and physical environments. From epigenetic modification to synaptic plasticity and activity-dependent myelination, mammalian brains develop and maintain their circuitry through ceaseless interaction with whatever environment they are immersed in [49, 50]. And for human beings, this environment is dominated by a complex social order in which gender is the foremost divisor [51]. No other fact about a new child flips more environmental switches than the so-called “gender reveal”. From names, clothes, toys, nursery décor, and pronouns [52] to the divergent communication styles, interactions, and expectations of parents [53], peers, and teachers [54], gender assignment bifurcates the experience of boys and girls from birth in every human culture, past and present [55]. This is especially pertinent given the older age of the UK Biobank and other large samples that have been widely used to elucidate normative sex/gender brain differences, since gender-differentiated experiences accumulate over the lifespan.

In fact, there is a competing theory addressing the biological origins of gender identity and expression. It is an extension of Esther Thelen’s [56] dynamic systems theory, and advances a more plastic, or soft-assembled mode of sex/gender development than brain organization theory. So rather than an explicit selection for different male and female brain structures, it posits that the primary selection in evolution was for large and highly plastic brains that permit the rapid and dynamic assembly of circuits most valued by the environment in which they are reared—which in most cultures includes strict adherence to gender roles [57]. Of course, one could argue that gender roles themselves served an adaptive role in human evolution, but from a neuroscience—and perhaps clinical—perspective, it matters whether brain sex/gender differentiation is the cause or consequence of these roles.

To their credit, DeCasien and colleagues do allude to the sociocultural origins of human sex/gender difference. First, by acknowledging the contribution of clinician gender bias toward disproportionate diagnoses of autism and major depression, and second, in acknowledging that “enduring socio-environmental factors could conceivably influence brain organization.”

This is important, especially given the end of their commentary in which they expound a firm commitment to anti-sexism. In this, I hope we can agree that a truly anti-sexist and intersectional neuroscience will recognize, first and foremost, that humans are remarkably diverse, and that our diverse and fluid identities and accompanying life experiences impact brain structure and connectivity. In other words, that any small sex/gender differences found using a specific set of methods in a certain Eurocentric database is unlikely to reflect a single, fixed “reality” that is true across humanity, with its rainbow of cultures, sexualities, and gender identities.

## Perspectives and significance

Recent policies in the U.S., Canada, and European Union have elevated sex/gender as a pre-eminent variable to be studied across biomedical research [58]. But when one attribute takes precedence over others as a matter of national policy, and entire journals are dedicated to publishing positive findings about it, the equipoise of scientific inquiry may be compromised. Sex/gender differences are unlike most biological findings in that they are widely consumed by the public [59, 60] and even applied to sociopolitical debates about who is male and who is female [61]. Recent large brain MRI studies have enormous statistical power to identify small male/female differences of questionable clinical or behavioral relevance. Scientists should exert greater caution in interpreting such modest and jittery findings in the context of humans’ non-binary and multifactorial gender behavioral differences and health disparities.

## Data Availability

Not applicable. All data described and tabulated have been published previously, as cited.

## Footnotes

1 For the remainder of this commentary, and following other recent usage (reviewed in [1]), the term “sex/gender” is used for the independent variable of interest, to connote the entangled influences of genetic and environmental factors on male–female brain and behavioral differences in humans. When referring specifically to animal or the combination of animal and human studies, the term “sex” will be used.

## References

[1] Eliot L, Ahmed A, Khan H, Patel J (2021). Dump the “dimorphism”: comprehensive synthesis of human brain studies reveals few male-female differences beyond size. Neurosci Biobehav Rev.

[2] Williams CM, Peyre H, Toro R, Ramus F (2021). Neuroanatomical norms in the UK Biobank: the impact of allometric scaling, sex, and age. Hum Brain Mapp.

[3] McDowell MA, Fryar CD, Ogden CL, Flegal KM. Anthropometric reference data for children and adults: United States, 2003–2006. Health Stat Rep. 2008; 1–48. 25585443

[4] de la Grandmaison GL, Clairand I, Durigon M (2001). Organ weight in 684 adult autopsies: new tables for a caucasoid population. Forensic Sci Int.

[5] Nottebohm F, Arnold AP (1976). Sexual dimorphism in vocal control areas of the songbird brain. Science.

[6] DeCasien AR, Guma E, Liu S, Raznahan A (2022). Sex differences in the human brain: a roadmap for more careful analysis and interpretation of a biological reality. Biol Sex Differ.

[7] Goldstein JM, Seidman LJ, Horton NJ, Makris N, Kennedy DN, Caviness VS (2001). Normal sexual dimorphism of the adult human brain assessed by in vivo magnetic resonance imaging. Cereb Cortex.

[8] Steen RG, Hamer RM, Lieberman JA (2007). Measuring brain volume by MR imaging: Impact of measurement precision and natural variation on sample size requirements. Am J Neuroradiol.

[9] Marek S, Tervo-Clemmens B, Calabro FJ, Montez DF, Kay BP, Hatoum AS (2022). Reproducible brain-wide association studies require thousands of individuals. Nature.

[10] Potvin O, Dieumegarde L, Duchesne S (2018). Corrigendum to ‘normative morphometric data for cerebral cortical areas over the lifetime of the adult human brain [NeuroImage 156 (2017) 315–339]’. Neuroimage.

[11] Potvin O, Dieumegarde L, Duchesne S (2017). Normative morphometric data for cerebral cortical areas over the lifetime of the adult human brain. Neuroimage.

[12] Lotze M, Domin M, Gerlach FH, Gaser C, Lueders E, Schmidt CO, Neumann N (2019). Novel findings from 2,838 adult brains on sex differences in gray matter brain volume. Sci Rep.

[13] Liu S, Seidlitz J, Blumenthal JD, Clasen LS, Raznahan A (2020). Integrative structural, functional, and transcriptomic analyses of sex-biased brain organization in humans. Proc Natl Acad Sci U S A.

[14] Ruigrok AN, Salimi-Khorshidi G, Lai MC, Baron-Cohen S, Lombardo MV, Tait RJ, Suckling J (2014). A meta-analysis of sex differences in human brain structure. Neurosci Biobehav Rev..

[15] Williams CM, Peyre H, Toro R, Ramus F (2021). Sex differences in the brain are not reduced to differences in body size. Neurosci Biobehav Rev.

[16] Kong XZ, Francks C, ENIGMA Laterality Working Group (2022). Reproducibility in the absence of selective reporting: an illustration from large-scale brain asymmetry research. Hum Brain Mapp.

[17] Despotović I, Goossens B, Philips W (2015). MRI segmentation of the human brain: challenges, methods, and applications. Comput Math Methods Med.

[18] Perlaki G, Horvath R, Nagy SA, Bogner P, Doczi T, Janszky J, Orsi G (2017). Comparison of accuracy between FSL’s FIRST and Freesurfer for caudate nucleus and putamen segmentation. Sci Rep.

[19] Zilles K, Kawashima R, Dabringhaus A, Fukuda H, Schormann T (2001). Hemispheric shape of European and Japanese brains: 3-D MRI analysis of intersubject variability, ethnical, and gender differences. Neuroimage.

[20] Ritchie SJ, Cox SR, Shen X, Lombardo MV, Reus LM, Alloza C, Harris MA, Alderson HL, Hunter S, Neilson E (2018). Sex differences in the adult human brain: evidence from 5216 UK Biobank participants. Cereb Cortex.

[21] Tan A, Ma W, Vira A, Marwha D, Eliot L (2016). The human hippocampus is not sexually-dimorphic: meta-analysis of structural MRI volumes. Neuroimage.

[22] Marwha D, Halari M, Eliot L (2017). Meta-analysis reveals a lack of sexual dimorphism in human amygdala volume. Neuroimage.

[23] Pintzka CWS, Hansen TI, Evensmoen HR, Håberg AK (2015). Marked effects of intracranial volume correction methods on sex differences in neuroanatomical structures: a HUNT MRI study. Front Neurosci.

[24] Hänggi J, Fovenyi L, Liem F, Meyer M, Jäncke L (2014). The hypothesis of neuronal interconnectivity as a function of brain size—a general organization principle of the human connectome. Front Hum Neurosci.

[25] Bush EC, Allman JM (2003). The scaling of white matter to gray matter in cerebellum and neocortex. Brain Behav Evol.

[26] de Jong LW, Vidal JS, Forsberg LE, Zijdenbos AP, Haight T, Sigurdsson S, Gudnason V, van Buchem MA, Launer LJ, Alzheimer’s Disease Neuroimaging Initiative (2017). Allometric scaling of brain regions to intra-cranial volume: an epidemiological MRI study. Hum Brain Mapp.

[27] Zhang K, Sejnowski TJ (2000). A universal scaling law between gray matter and white matter of cerebral cortex. Proc Natl Acad Sci.

[28] Sommer IE, Aleman A, Somers M, Boks MP, Kahn RS (2008). Sex differences in handedness, asymmetry of the planum temporale and functional language lateralization. Brain Res.

[29] Hirnstein M, Hugdahl K, Hausmann M (2019). Cognitive sex differences and hemispheric asymmetry: a critical review of 40 years of research. Laterality.

[30] Sato M (2020). The neurobiology of sex differences during language processing in healthy adults: a systematic review and a meta-analysis. Neuropsychologia.

[31] David SP, Naudet F, Laude J, Radua J, Fusar-Poli P, Chu I, Stefanick ML, Ioannidis JP (2018). Potential reporting bias in neuroimaging studies of sex differences. Sci Rep.

[32] Rich-Edwards JW, Kaiser UB, Chen GL, Manson JE, Goldstein JM (2018). Sex and gender differences research design for basic, clinical, and population studies: essentials for investigators. Endocr Rev.

[33] Garcia-Sifuentes Y, Maney DL (2021). Reporting and misreporting of sex differences in the biological sciences. Elife.

[34] Ricard JA, Parker TC, Dhamala E, Kwasa J, Allsop A, Holmes AJ (2022). Confronting racially exclusionary practices in the acquisition and analyses of neuroimaging data. Nat Neurosci.

[35] Joel D, Persico A, Salhov M, Berman Z, Oligschläger S, Meilijson I, Averbuch A (2018). Analysis of human brain structure reveals that the brain “types” typical of males are also typical of females, and vice versa. Front Hum Neurosci.

[36] Yang G, Bozek J, Han M, Gao JH (2020). Constructing and evaluating a cortical surface atlas and analyzing cortical sex differences in young Chinese adults. Hum Brain Mapp.

[37] Fine C (2017). Testosterone rex: myths of sex, science, and society.

[38] Laland K, Brown G (2011). Sense and nonsense: evolutionary perspectives on human behavior.

[39] Tang-Martínez Z (2016). Rethinking Bateman's principles: challenging persistent myths of sexually reluctant females and promiscuous males. J Sex Res..

[40] Haas R, Watson J, Buonasera T, Southon J, Chen JC, Noe S, Smith K, Llave CV, Eerkens J, Parker G (2020). Female hunters of the early Americas. Sci Adv..

[41] Anderson A, Chilczuk S, Nelson K, Ruther R, Wall-Scheffler C (2023). The myth of man the hunter: women's contribution to the hunt across ethnographic contexts. PLoS One..

[42] Jordan-Young RM (2011). Brain storm: the flaws in the science of sex differences.

[43] Xiong H, Peterson JB, Scott S (2020). Amniotic testosterone and psychological sex differences: a systematic review of the extreme male brain theory. Dev Rev.

[44] Khalifeh N, Omary A, Cotter DL, Kim MS, Geffner ME, Herting MM (2022). Congenital adrenal hyperplasia and brain health: a systematic review of structural, functional, and diffusion magnetic resonance imaging (MRI) investigations. J Child Neurol.

[45] Vijayakumar N, de Macks ZO, Shirtcliff EA, Pfeifer JH (2018). Puberty and the human brain: insights into adolescent development. Neurosci Biobehav Rev.

[46] Comasco E, Frokjaer VG, Sundström-Poromaa I (2014). Functional and molecular neuroimaging of menopause and hormone replacement therapy. Front Neurosci.

[47] Frizell B, Dumas JA (2018). Examining the relationship between neurosteroids, cognition, and menopause with neuroimaging methods. Curr Psychiatry Rep.

[48] Hines M, Ahmed SF, Hughes IA (2003). Psychological outcomes and gender-related development in complete androgen insensitivity syndrome. Arch Sex Behav.

[49] Lindenberger U, Lövdén M (2019). Brain plasticity in human lifespan development: the exploration–selection–refinement model. Annu Rev Dev Psychol.

[50] Zatorre RJ, Fields RD, Johansen-Berg H (2012). Plasticity in gray and white: neuroimaging changes in brain structure during learning. Nat Neurosci.

[51] Weisman K, Johnson MV, Shutts K (2015). Young children's automatic encoding of social categories. Dev Sci.

[52] Zosuls KM, Ruble DN, Tamis-LeMonda CS, Shrout PE, Bornstein MH, Greulich FK (2009). The acquisition of gender labels in infancy: Implications for gender-typed play. Dev Psychol.

[53] Mesman J, Groeneveld MG (2018). Gendered parenting in early childhood: subtle but unmistakable if you know where to look. Child Dev Perspect.

[54] Kollmayer M, Schober B, Spiel C (2018). Gender stereotypes in education: development, consequences, and interventions. Eur J Dev Psychol.

[55] Whiting BB, Edwards CP (1988). Children of different worlds: the formation of social behavior.

[56] Thelen E (2005). Dynamic systems theory and the complexity of change. Psychoanal Dialogues.

[57] Fausto-Sterling A (2021). A dynamic systems framework for gender/sex development: from sensory input in infancy to subjective certainty in toddlerhood. Front Hum Neurosci.

[58] White J, Tannenbaum C, Klinge I, Schiebinger L, Clayton J (2021). The integration of sex and gender considerations into biomedical research: lessons from international funding agencies. J Clin Endocrinol Metab.

[59] Eliot L (2011). The trouble with sex differences. Neuron.

[60] Maney DL (2015). Just like a circus: the public consumption of sex differences. Curr Top Behav Neurosci.

[61] Sudai M, Borsa A, Ichikawa K, Shattuck-Heidorn H, Zhao H, Richardson SS (2022). Law, policy, biology, and sex: critical issues for researchers. Science.

